# The safety and effectiveness of heated humidified high-flow nasal cannula as an initial ventilation method in the treatment of neonatal respiratory distress syndrome: A protocol for systematic review and meta-analysis

**DOI:** 10.1097/MD.0000000000023243

**Published:** 2020-11-13

**Authors:** Shu-Ni Li, Li Li, Chun-Lei Li, Shu-Ping Zhou, Wei-Cheng Lu

**Affiliations:** a Department of Nursing; b Department of Pediatrics Area One; c Department of Neonatology, Hainan General Hospital (Hainan Affiliated Hospital of Hainan Medical University), NO.19 Xiuhua Road, Xiuying District, Haikou, Hainan, PR China.

**Keywords:** heated humidified high-flow nasal cannula, initial ventilation method, nasal continuous positive airway pressure, neonatal respiratory distress syndrome, pulmonary surfactant, randomized controlled trial, systematic review and meta-analysis

## Abstract

**Background:**

This study uses a method of systematic evaluation to evaluate the safety and effectiveness of heated humidified high-flow nasal cannula (HHHFNC) as an initial ventilation method in the treatment of neonatal respiratory distress syndrome (NRDS) scientifically. In the field of evidence-based medicine, this study provides a theoretical reference and basis for choosing appropriate initial non-invasive ventilation methods in the treatment of NRDS, thereby providing assistance for clinical treatment.

**Methods:**

The main electronic network databases were searched by computer, including 4 Chinese databases: CNKI, WangFang Data, CQVIP, SinoMed and 3 English databases: PubMed, The Cochrane Library and EMBASE, the time range of retrieval from the beginning of each database to September 1, 2020. The content involves all the published randomized controlled trials on the effectiveness of HHHFNC compared with NCPAP as an initial ventilation method in the treatment of NRDS. Using a search method that combines medical subject words and free words. Based on the Cochrane risk bias assessment tool, 2 researchers independently screen the literature, and then extract the data we needed in the literature, and cross-check. If it is difficult to decide whether to include literature, then turning to a third researcher for help and making a final decision after discussion, and using RevMan 5.3 and STATA 13.0 to analyze the relative data.

**Results:**

Based on the method of meta-analysis, this study analyzes the pre-determined outcome indicators through scientific statistical analysis, and compares the effectiveness and safety of HHHFNC compared with NCPAP as an initial ventilation method in the treatment of NRDS. All results will be published in peer-reviewed high-quality professional academic journals.

**Conclusion:**

Based on evidence-based medicine, this study will obtain the establishing evidence of comparison that the clinical effectiveness and safety of HHHFNC compared with NCPAP as an initial ventilation method in the treatment of NRDS through the existing data and data, which provides the evidence support of evidence-based medicine in the treatment of NRDS.

**OSF registration number:**

September 17, 2020. osf.io/f6at4 (https://osf.io/f6at4).

## 1 Introduction

With the development of perinatology, the survival rate of very low/ultra-low premature infants of gestational age is increasing gradually, but the diseases that threaten the life safety of premature infants are also increasing. In particular, the incidence of neonatal respiratory distress syndrome (NRDS) is increasing year by year, NRDS has become one of the important risk factors that threaten early survival and late quality of life of premature infants.^[1–4]^ NRDS is common in premature infants and selective cesarean section of the newborn, which refers to the cyanosis of a newborn shortly after birth, and the progressive exacerbation of dyspnea, even the disease progresses to respiratory failure, because of its pathological appearance of pulmonary hyaline membrane, it is also called hyaline membrane disease (HMD).^[5]^

The etiology of NRDS is complicated. It is generally recognized that NRDS is due to the lack of pulmonary surfactant (PS), and the increase of alveolar surface tension leads to pulmonary edema and progressive alveolar atrophy.^[6]^ The typical clinical symptoms of NRDS are mainly found in premature infants, and it can appear cyanosis and shortness of breath within 1–2 hours after birth, and progressive dyspnea, groaning, inspiratory 3 concave sign is positive, etc. The clinical symptoms will be very obvious 6 hours after birth, even irregular breathing, apnea, respiratory failure. Clinical studies have shown that: the incidence of NRDS is related to gestational age, weight and so on. The smaller the gestational age and the lower the weight, the higher the morbidity and mortality.^[7]^ Some studies have shown that NRDS is a self-limited disease, with the maturation of lung and the increase of PS synthesis, the clinical symptoms of most children will gradually improve in the third day after birth.^[8]^ Due to the rapid progress of the disease, if it can not be treated in time, it is often accompanied by hypoxemia and pulmonary edema, which leads to the formation of hyaline membrane of lung and leads to poor prognosis, so it is of the main causes of neonatal death. Therefore, it is very important to find suitable, effective and safe treatments.^[9]^

The treatment of NRDS includes general treatment, respiratory support and PS replacement therapy, in respiratory support, non-invasive ventilation has become the first clinical choice of ventilation. There is a great variety of non-invasive ventilation, the classic ones are NCPAP, nasal biphasic positive airway pressure (NBIPAP), nasal intermittent positive pressure ventilation (NIPPV), which are derived from it, noninvasive synchronized intermittent mandatory ventilation (n-SIMV), HHHFNC and so on.^[10]^ For the children with NRDS, the European NRDS treatment guidelines proposed that the best treatment scheme for NCPAP combined with early pulmonary surfactant therapy in 2016. NCPAP provides constant airflow and airway pressure for children during the entire using process by setting the parameters.^[11]^ It is one of the commonly used non-invasive ventilation methods in neonatal intensive care unit (NICU). With the increase of clinical use of NCPAP, its related shortcomings are gradually exposed. NCPAP usually use nasal mask to ventilate, and the nasal congestion is not easy to fix, and it is easy to cause nasal congestion to fall off when children are active. Therefore, it is inconvenient for nursing to wear a special hat and maintain the body position to maintain the fixation; it increases the discomfort of the children, and due to the increase of crying, excessive gas enters the gastrointestinal tract and causes abdominal distension. The long-term compression of nasal congestion and hat can cause skin breakage and even necrosis around the nose, obvious expansion of nostrils, nasal septum bias and head deformation.^[12,13]^

The medium of HHHFNC is nasal catheter, and it is simple to operate and convenient in nursing, so it is gradually valued by clinicians as an alternative ventilation mode of NCPAP. HFNC ventilation was first used in clinical treatment of RDS in 1992, and the gas was not heated and humidified at the beginning. But in the process of time, its clinical using experience is increasing, and the using technology is maturing, then it gradually develops into HHHFNC. By heating the input gas (generally heated to approximately 37°C of human temperature), reducing the damage and stimulation of respiratory mucosa by air conditioning flow and maintaining the integrity of the respiratory tract, preventing bronchospasm, improving lung compliance, ensuring its ventilation and ventilation function, but also maintaining body temperature, and reducing the loss of heat. The humidification of gas can prevent the dryness of airway, protect the swing function of airway cilia, enhance the defense ability of airway to external microorganisms, promote the discharge of secretions, prevent the airway blockage, keep unobstructed, and reduce the loss of water, and prevent the insufficient liquid.^[14,15]^

The use of HHHFNC in clinical diseases has become more and more popular because of its many advantages. In recent years, many studies have applied HHHFNC as the initial ventilation mode of NRDS children in clinical practice, but most of them are small sample studies, the clinical evidence is limited. Comparing with traditional NCPAP, its safety and effectiveness are still controversial. This study conducts a systematic review and meta-analysis to the randomized controlled trial (RCT) on the effectiveness and safety of HHHFNC and NCPAP as initial ventilation methods in the treatment of NRDS at home and abroad, which provides help for the clinical treatment of the disease from evidence-based medicine.

## 2 Methods

### 2.1 Protocol and registration

The protocol has been registered in the Open Science Framework (OSF) and the OSF registration number: September 17, 2020. osf.io/f6at4. (https://osf.io/f6at4).

### 2.2 Inclusion criteria

#### 2.2.1 Study type

This study will collect the RCT of HHHFNC ventilation compared with NCPAP as an initial ventilation method in the treatment of NRDS. The language and publication status of the literature are not restricted.

#### 2.2.2 Types of patients

Newborns with respiratory distress syndrome who have symptoms of distress shortly after birth, such as exhalation groaning, superficial breathing, three-concave sign, and chest collapse. Newborns, premature and full-term infants who meet the NRDS diagnosis by chest X-ray or are clinically diagnosed as NRDS were included. The sex, gestational age, weight and condition of the children are comparable.

#### 2.2.3 Inclusion criteria

1. it refers to the diagnostic criteria for neonatal NRDS in the 2013 edition of the European guidelines for the management of NRDS, and including the patients who meet the criteria;^[16]^
2. acute onset, with groaning, progressive exacerbation of breathing, cyanosis, rib depression and other symptoms and progressive exacerbation;
3. when children inhaled air, PaO_2_<50 mm Hg and/or 50 mm Hg, and it needs oxygen to maintain PaO_2_>50 mm Hg or appears central bruising;
4. X-ray chest radiographs show that the lungs of grade I are generally low transmittance, fine particles and reticular shadows, grade II lungs are larger denser particles, reticular shadows and bronchiectasis, and grade III ground glass changing or bronchiectasis, grade IV white lungs are blurred heart shadow, and bronchial inflation sign is obvious.^[17,18]^

### 2.3 Exclusion criteria

1. other diseases that cause respiratory distress;
2. congenital diaphragmatic hernia, trachea-esophageal fistula, and abdominal cleft that require surgical treatment;
3. congenital respiratory malformations, cleft lip and palate, and mandibulofacial dysostosis and pneumothorax;
4. uncontrolled air leak syndrome, meconium aspiration syndrome, pulmonary hemorrhage, and grade III to IV intracranial hemorrhage;
5. outcome indicators are not described in the results of the study, or the research that the results are unknown or vague description;
6. the same research has been published repeatedly;
7. the language of the research report is not Chinese or English;
8. the research results cannot be obtained, the research data cannot be extracted, and the full text of the research cannot be found.

### 2.4 Intervention type

#### 2.4.1 Experimental group

Using HHHFNC ventilation combined with conventional treatment.

#### 2.4.2 Control group

Using NCPAP ventilation combined with conventional treatment.

### 2.5 Outcomes

#### 2.5.1 Main outcome indicators

1. The time of non-invasive ventilation;
2. the analysis of arterial blood gas;
3. the relief time of shortness of breath;
4. the failure rate of ventilation;
5. the exposure time of oxygen;
6. Feed time;
7. reach to the feeding time of whole intestine.

#### 2.5.2 Secondary outcomes

1. The incidence of complications (nasal injury, abdominal distension, air leakage, bronchopulmonary dysplasia, neonatal necrotizing enterocolitis, neonatal intracranial hemorrhage);
2. mortality rate;
3. total hospitalization time;
4. total hospitalization expenses.

### 2.6 Search strategy

Search strategy Using the computer to search common Chinese databases, including: CNKI, WanFang Data, CQVIP, SinoMed; common English databases, including: PubMed, The Cochrane Library and EMBASE. The time range of retrieval from the beginning of Chinese and English database to September 1, 2020. The language of the literature is set to Chinese or English. Using a search method that combines medical subject words and free words, and using Boolean logic operators (AND, OR, NOT) to perform combined search, and making corresponding adjustments to search for relevant documents as comprehensively as possible according to the search methods of different databases. At the same time, arranging researchers to search relevant literature and relevant conference paper abstracts of pediatrics, neonatal science, and neonatal nursing in the past 10 years through manual search, and tracing the relevant literature to avoid omissions based on their references. If necessary, obtaining more detailed information about the relevant research by contacting the corresponding author by post. Searching the PubMed database as an example, the search strategy is shown in Table 1.

**Table 1:** Search strategy used in PubMed database.

No	Strategy
1	Respiratory Distress Syndrome, Newborn [Mesh Terms]
2	(Respiratory Distress Syndrome, Newborn [Title/Abstract]) OR (Neonatal Respiratory Distress Syndrome [Title/Abstract]) OR (Respiratory Distress Syndrome, Infant [Title/Abstract]) OR (Infantile Respiratory Distress Syndrome [Title/Abstract])
3	Noninvasive Ventilation[Mesh Terms]
4	(Noninvasive Ventilation [Title/Abstract]) OR (Noninvasive Ventilations [Title/Abstract]) OR (Ventilation, Noninvasive [Title/Abstract]) OR (Ventilations, Noninvasive [Title/Abstract]) OR (Non-Invasive Ventilation [Title/Abstract]) OR (Non-Invasive Ventilations [Title/Abstract]) OR (Ventilation, Non-Invasive [Title/Abstract]) OR (Ventilations, Non-Invasive [Title/Abstract]) OR (Non Invasive Ventilation [Title/Abstract]) OR (Non Invasive Ventilations [Title/Abstract]) OR (Ventilation, Non Invasive [Title/Abstract]) OR (Ventilations, Non Invasive [Title/Abstract])
5	Heated Humidified High Flow Nasal Cannula [Mesh Terms]
6	(Heated Humidified High Flow Nasal Cannula [Title/Abstract]) OR (Humidified High Flow Nasal Cannula [Title/Abstract]) OR (High-Flow Nasal Cannula [Title/Abstract])
7	Continuous Positive Airway Pressure [Mesh Terms]
8	(Continuous Positive Airway Pressure [Title/Abstract]) OR (CPAP Ventilation [Title/Abstract]) OR (Ventilation, CPAP [Title/Abstract]) OR (Biphasic Continuous Positive Airway Pressure [Title/Abstract]) OR (Bilevel Continuous Positive Airway Pressure [Title/Abstract]) OR (Nasal Continuous Positive Airway Pressure [Title/Abstract]) OR (nCPAP Ventilation [Title/Abstract]) OR (Ventilation, nCPAP [Title/Abstract]) OR (Airway Pressure Release Ventilation [Title/Abstract]) OR (APRV Ventilation Mode [Title/Abstract]) OR (APRV Ventilation Modes [Title/Abstract]) OR (Ventilation Mode, APRV [Title/Abstract]) OR (Ventilation Modes, APRV [Title/Abstract])
9	Randomized Controlled Trial [Publication Type]
10	Randomized Controlled Trial [Title/Abstract]
11	1 OR 2
12	3 OR 4
13	5 OR 6
14	7 OR 8
15	9 OR 10
16	12 AND 13 AND 14
17	11 AND 15 AND 16

Table 1. Retrieval strategies of PubMed.

### 2.7 Selection process

Two researchers (SL, LL) independently reviewed the literature, and extract the data and cross-check, if differences, through discussion or consultation with third parties to resolve, the lack of information to contact the author to supplement as far as possible. Reading the title and abstract at first in the process of literature screening, and reading the full text further to determine whether it is finally included after excluding significantly unrelated literature. If necessary, the studies that provide only median, range, or interquartile spacing in the results convert the effect quantity to mean and standard deviation.^[19]^ Data extraction includes the following aspects:

1. the basic information of inclusion study, including the research topic, the first author, the year of publication, the country of publication and the published journal, etc.;
2. the baseline characteristics of the subjects, including the sample size, the gender, age of patient and intervention;
3. the specific details of the intervention.

**Figure 1:**
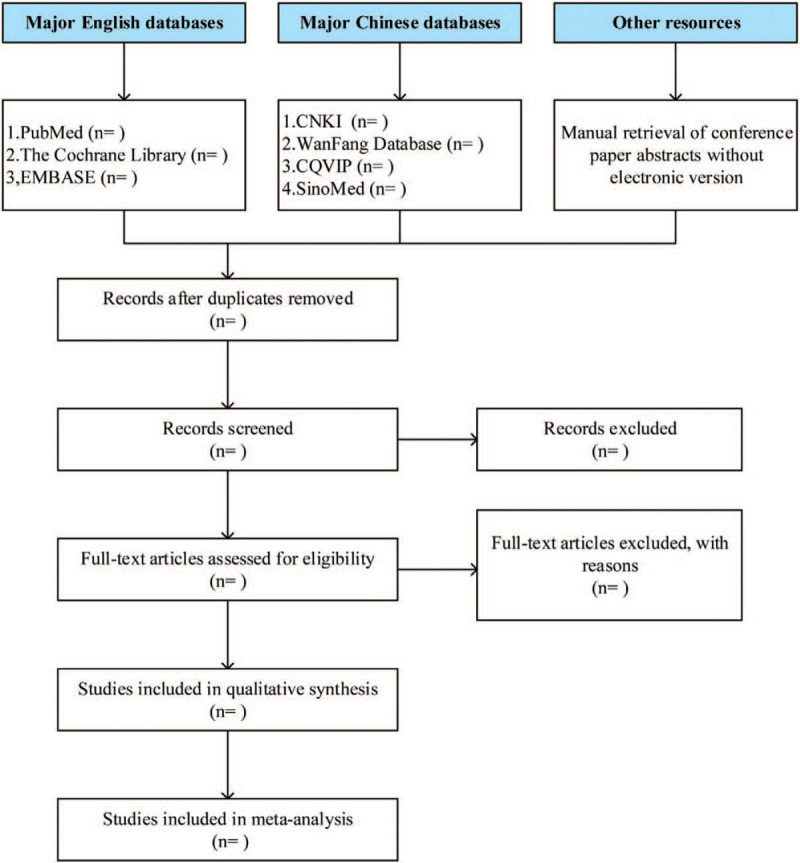
Study selection flow diagram.

Treatment of missing values: obtaining raw data is still a gold standard for processing missing data, but when the original research does not provide sufficient data and it can not be obtained through other channels, the estimation method is still very practical to deal with the missing values in the research results. During the use of the above defects, it is suggested that some stability test be carried out to observe whether these estimates and transformations will have a significant impact on the overall results.^[20]^ The results of study selection flow diagram are shown in Figure 1.

### 2.8 Risk of bias assessment

The quality of included study is evaluated with reference to the relevant criteria recommended in the Cochrane systematic evaluation manual 5.0, which mainly includes 6 aspects, including whether the specific scheme is random, whether there is allocation hiding, whether blind method is used (blind method includes 2 aspects, blind method for the study subjects and blind method for the study implementer or outcome measures), whether the outcome index is complete, whether the outcome index is unclear or missing, whether there is selective reporting of the results and whether there are other sources of bias. Three kinds of judgments are made for the above items: “Yes” (reports a low degree of bias), “No” (reports a high degree of bias), and “Unclear” (reports an unknown degree of bias). Among them, the more results are judged as “yes”, indicating that the higher the quality of the study, the less likely it is to be biased.^[21]^

### 2.9 Data analysis

Using RevMan 5.3 software for meta-analysis. The WMD and 95% CI are used as the effect quantity for the continuous data. Using Chi-Squared test to analyze the heterogeneity among the results of the study, and judging the heterogeneity by *I*^2^. When the *P* > is .1 and the *I*^2^ ≤ 50%, it is suggested that there is no statistical heterogeneity or the heterogeneity is small among the studies, and using the fixed effect model to analyze it; conversely, it is suggested that there is a statistical heterogeneity among the studies, and using random effect model to analyze. For obvious clinical heterogeneity, subgroup analysis or sensitivity analysis or only descriptive analysis is used, and the test level of meta-analysis is α = 0.05.

### 2.10 Subgroup analysis

For the problem of large heterogeneity of some outcome indicators in the study, subgroup analysis can be used as an effective means. Subgroup analysis is to divide the studies into 2 or more groups according to one of the above factors, to observe whether the difference among the effect quantity is statistically significant after combining the effect. That is, whether there is interaction between subgroup combined effect and grouping factor, which determines whether the grouping factors are the important contributor factor for the heterogeneity between the research results.^[22]^ Grouping factor can be the factors, which not directly related to the evaluation of the efficacy of intervention. This study will carry out subgroup analysis to the outcome indicators with greater heterogeneity by related objective factors such as gestational age, weight, duration of treatment, region, etc, so as to reduce the heterogeneity of outcome indicators.

### 2.11 Sensitivity analysis

Deleting the included studies one by one, observing the changes of effect value and heterogeneity before after the deletion, so as to evaluate whether each study will have an impact on the overall results. If the heterogeneity changes after deleting a study and the combined effect value is still statistically significant, it is considered that the study has heterogeneity. It is necessary to further analyze the sources of heterogeneity, such as the number of samples, whether the original data included in the study is accurate, whether the method of extracting data is correct, and so on. If the combined effect value and the changes of heterogeneity are not significant after the deletion, then it shows that the results are reliable and robust.

### 2.12 Publication bias

The funnel chart of RevMan 5.3 software is often used for subjective identification of publication bias. Generally, when the number of research articles is ≥7, the publication bias of the research is studied in the form of a funnel chart. If the data is biased, it will appear an asymmetric funnel chart, and the more obvious the asymmetry, the greater the degree of bias. In addition, the Egger testing method of using STATA 13.0 software can be used to objectively analyze bias. When *P* > .05, it can be considered that there is no publication bias, otherwise, there is publication bias.

### 2.13 Ethics and dissemination

This study belongs to the category of systematic review and it is only a secondary analysis of the published data, so ethical approval is not applicable to this study.

## 3 Discussion

As a traditional ventilation mode, NCPAP is often the first choice for children with NRDS who do not need invasive respiratory support. NCPAP can provide a certain amount of airway pressure to correct or reverse the collapse of alveolus, restore sufficient functional residual air volume, prevent the progression of the disease, maintain the function of alveolus, and transport a certain amount of oxygen to promote oxygenation, improve hypoxia and respiratory distress; it can also reduce the inactivation of PS to a certain extent, promote its production, and correct the internal factors of respiratory distress. In severe cases, the effectiveness is better when combined with exogenous PS. However, the traditional NCPAP is inconvenient to care, due to the use of masks and nasal congestion, and it may cause certain damage to children, which will affect the clinical rehabilitation of children. Therefore, it has become the desire of the majority of neonatology clinicians to seek a non-invasive ventilation mode with less damage to children, convenient operation, and no less safety and effectiveness than NCPAP.^[23]^ HHHFNC originated from nasal catheter oxygen therapy, its equipment is simple, and it is easy to operate and care, the used nasal catheter is small in size, light in weight, and it is not easy to cause compression on local tissues, nasal septum bias and nostril expansion. The children are also easy to tolerate, and it has high comfort, and when the gas flow is sufficient, it can produce similar CPAP effect.^[24]^ The mechanism of action includes:

1. high flow gas can produce a certain positive airway pressure, and it is similar to NCPAP, which can prevent the collapse of airway, promote lung recruitment, and maintain the function of alveolus;
2. by the scouring effect of high flow gas, promoting the removal of residual CO2 from nasopharynx, reducing the dead cavity ventilation, increasing the alveolus ventilation volume, improving the efficiency of lung ventilation;
3. the heated gas can reduce the damage and stimulation of cold air flow to respiratory tract mucosa, prevent bronchospasm, improve lung compliance, maintain body temperature and reduce the loss of heat;
4. the humidified gas can protect the airway cilia, enhance the defense ability of the airway to the external microorganism, prevent the airway from drying, promote the discharge of respiratory secretions, and keep the respiratory tract unobstructed;
5. through the nasal catheter directly to transport a certain flow of gas after heating, humidification, it can also reduce the work of breathing, reduce the resistance of the upper airway during inhalation to achieve auxiliary ventilation.

As an initial ventilation mode in the treatment of children with NRDS, HHHFNC is still in the exploration stage. In recent years, many clinical studies comparing the 2 groups of ventilation methods have been published, and their conclusions are also different and controversial. Moreover, most of the studies have small sample size, which is not of sufficient clinical significance.^[25]^

The significance of this study is that the safety and effectiveness of HHHFNC compared with traditional NCPAP are still controversial. Therefore, this study will give a positive conclusion by systematically evaluating the effectiveness and safety of HHHFNC and NCPAP in the treatment of NRDS as initial ventilation at home and abroad, which to provide theoretical support and help for the clinical treatment of NRDS.

## Author contributions

**Conceptualization:** Shu-Ni Li, Wei-Cheng Lu.

**Formal analysis:** Shu-Ni Li, Li Li.

**Funding acquisition:** Wei-Cheng Lu.

**Methodology:** Shu-Ni Li, Chun-Lei Li.

**Software:** Li Li, Shu-Ping Zhou.

**Supervision:** Wei-Cheng Lu.

**Writing – original draft:** Shu-Ni Li, Li Li, Chun-Lei Li, Shu-Ping Zhou.

**Writing – review & editing:** Shu-Ni Li, Wei-Cheng Lu.
